# Export priority technique for Uni-portal thoracoscopic left upper lobectomy

**DOI:** 10.1186/s13019-024-02738-3

**Published:** 2024-05-03

**Authors:** Yanhui Yang, Ji Li, Xin Cheng, Sipeng Cheng, Xiaoyang Xie

**Affiliations:** Department of Cardiothoracic Surgery, The First People’s Hospital of Neijiang, 1866, West Section of Hanan Avenue, Shizhong District, Neijiang, Sichuan 641000 China

**Keywords:** UVATS, Left upper lobe, Export priority technique

## Abstract

**Background:**

Further explore the safety and feasibility of Uni-portal video assisted thoracoscopic (UVATS) left upper lobectomy by optimizing the treatment of incisions and blood vessels.

**Methods:**

We conducted a retrospective analysis of data from 32 patients who underwent UVATS left upper lobectomy and systematic mediastinal lymph node dissection utilizing the Export priority technique between January 2021 and December 2022. We documented perioperative indicators, including surgical time, intraoperative blood loss, the number of lymph nodes dissected, and postoperative pathological staging.

**Results:**

All surgeries were conducted utilizing the Export priority technique in UVATS. The mean surgical duration was (98.93 ± 14.98) minutes, with an average intraoperative blood loss of (79.53 ± 37.96) ml. The mean count of dissected lymph nodes was (13.96 ± 2.69). The length of hospital Stay averaged (5.62 ± 1.81) days. On the first postoperative day, the thoracic drainage volume was (101.87 ± 49.46) ml. The mean duration of postoperative thoracic tube insertion was (3.1 ± 1.84) days. No occurrences of postoperative hoarseness, pulmonary infection, or complications such as bronchopleural fistula were observed.

**Conclusion:**

The application of the Export priority technique improves the safety and feasibility of UVATS left upper lobectomy.

## Introduction

As thoracoscopic instruments and technology advance swiftly, the surgical approach has evolved from the initial three-hole and double-hole methods to the present Uni-portal approach [1, 2]. This modern approach allows the entire surgery to be conducted through a 3-4 cm incision on the chest wall [3, 4]. Currently, the majority of Scholars advocate selecting the UVATS surgical incision at the 4th or 5th intercostal space [5–7]. Precision in selecting the optimal incision is imperative to enhance the surgeon's experience and ensure the overall safety of the surgical procedure. Nevertheless, in the case of obese patients, the increased thickness of subcutaneous adipose tissue poses challenges to rib counting and positioning. Effectively addressing this, the swift and accurate localization of the incision becomes the initial crucial step for surgeons in achieving successful surgery.

UVATS minimally invasive surgery comprehensively addresses a wide spectrum of thoracic diseases, contributing to a significant enhancement in the long-term quality of life for patients post-surgery [8–11]. UVATS left upper lobectomy is often considered the most challenging with a higher risk of bleeding in minimally invasive lobectomy, primarily attributed to the following factors: Firstly, the left upper lobe of the lung contains numerous blood vessels with a high likelihood of variation, especially the left upper pulmonary artery, encompassing LA^1+2^a + b, A^1+2^c, LA^3^, LA^4^, LA^5^. Secondly, the left lung tissue features oblique fissures, and inadequate development of these fissures complicates arterial treatment via the interlobular fissure pathway, introducing challenges to the surgical process. Thirdly, the intricate arrangement of pulmonary veins and arteries, along with the deep positioning of the left upper lobe bronchus amidst blood vessels, prohibits direct severance, eliminating potential surgical shortcuts. Fourthly, the presence of the mediastinal lingual artery may elevate the risk of bleeding. Effectively addressing these challenges under UVATS conditions to reduce postoperative complications has been a persistent concern for surgeons. To tackle these issues and enhance the safety and feasibility of Uni-portal thoracoscopic left upper lobectomy, we propose a novel concept that primarily focuses on optimizing incision treatment and Export priority technique. This concept may offer valuable insights for future clinical practice, especially considering the current lack of systematic and modular reports on Uni-portal left upper lobectomy.

## Method and materials

### Clinical summery

We retrospectively analyzed clinical case data from 32 patients who underwent Uni-portal thoracoscopic left upper lung resection and lymph node dissection at the Department of Thoracic Surgery, First People's Hospital of Neijiang City, between February 2021 and December 2022. Preoperative clinical variables recorded included age, gender, smoking history, BMI (Body Mass Index), FEV1 (Forced expiratory volume in 1 s), PO2 (Partial pressure of oxygen), Clinical TNM stage, history of malignancy, comorbidity, and maximum diameter of tumor, as outlined in Table 1. Perioperative clinical variables, such as pathologic staging, pathologic types, intraoperative bleeding volume, surgery time, number of lymph node dissections, postoperative thoracic tube insertion time, day 1 chest tube drainage, postoperative complications, and length of hospital stay, are detailed in Table 2.

**Table 1:** Preoperative clinical variables

Preoperative clinical variables	N
Gender	
Male	12(37.50%)
Female	20(62.50%)
Age(years)	52.53 ± 12.66
Smoking	
Yes	11(34.38%)
No	21(65.62%)
BMI	21.37 ± 3.57
FEV1(L)	1.89 ± 0.41
PO2(mmHg)	80.43 ± 6.57
Maximum diameter of tumor(cm)	2.59 ± 0.70
Comorbidity	
Hypertension	6
Diabetes	5
COPD	3
Asthma	1
Others	1
History of malignancy	0
Yes	7(21.87%)
NO	25(78.13%)
Clinical TNM stage	
I	18(56.25%)
II	12(37.5%)
IIIA	2(6.25%)

**Table 2:** Perioperative clinical variables

Perioperative clinical variables	N
Pathologic staging	
I	18(56.25%)
II	11(34.38%)
IIIA	3(9.36%)
Pathologic types	
Adenocarcinoma	19(59.37%)
Squamous cell carcinoma	9(28.12%)
Others	4(12.50%)
Intraoperative bleeding volume (ml)	79.53 ± 37.96
Surgical time(min)	98.93 ± 14.98
Number of lymph node dissection (pieces)	13.96 ± 2.69
Postoperative thoracic tube insertion time (day) Day 1, chest tube drainage (ml)	3.1 ± 1.84 101.87 ± 49.46
Hospital stay (day)	5.62 ± 1.81
Postoperative complications	
Bronchopleural fistula	0
Arrhythmias	0
chylothorax	1
Postoperative progressive hemothorax	0

#### Selection criteria

1). Chest CT or PET/CT（Positron emission tomography / Computed tomography）indicating a mass in the left upper lung and undergoing UVATS left upper lobectomy + lymph node dissection. 2). Surgery that conforms to the principle of prioritizing Exports 3). Clinical TNM staging is early or late stage (T1-3N0-2M0), with no invasion of the left lower lobe and surrounding tissues 4) Complete case data. 

#### Exclusion criteria

1). Patients with two or three orifices and transitioning to thoracotomy during surgery. 2). Intraoperative exploration revealed severe calcification and fusion of lymph nodes in the hilum and mediastinum of the lungs 3). Preoperative neoadjuvant therapy. 4). Patients with poor cardiovascular and pulmonary function who cannot tolerate surgery.

### Surgical technical

#### Preoperative preparation and incision

**Fig. 1:**
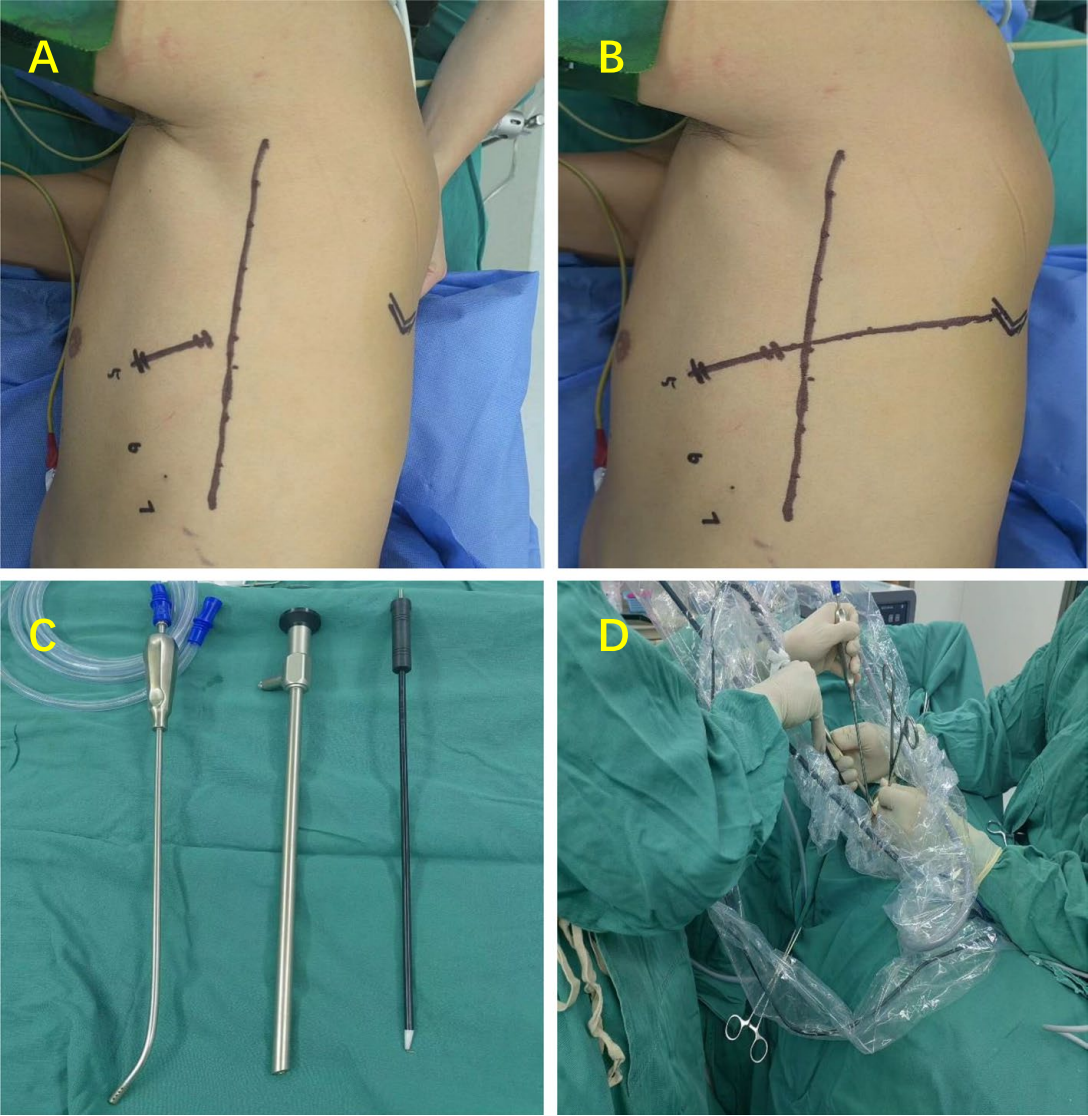
**A**, **B** The parallel positioning method for the inferior angle of the shoulder armor first marks the position of the inferior angle of the shoulder armor, and the parallel corresponding method can be used to quickly mark the 5th rib gap. **C** The conventional instruments for Uni-pot thoracoscopy, from left to right, are elbow suction device, 30°Storz thoracoscope, and electrocoagulation hook in sequence. **D** Two surgeons can complete the surgery, with the main knife located on the patient's ventral side and the assistant located on the patient's dorsal side. Assistant with right hand holding mirror, left hand exposing field of view.

Surgical fixation is executed by the same group of physicians employing dual-lumen tracheal intubation for general anesthesia and a single-lung ventilation mode. The patient is positioned laterally at a 90° angle, and to further widen the intercostal space, a soft pillow can be horizontally placed at the level of both nipples. Commencing with marking the inferior angle of the shoulder nail, a 3 cm incision is made on the anterior 5th intercostal plane along the axillary midline. Figure 1A, B). A disposable incision protective cover is inserted, and a 30° Storz thoracoscope is utilized with the endoscope body secured behind the incision using a 14# T-tube. Positioned on the patient's ventral side, the surgeon's supporting hand is on the dorsal side, with one hand supporting and the other wielding double-joint forceps to expose the surgical field. In the left hand, the surgeon holds an elbow suction device, while energy instruments such as electrocoagulation hooks or ultrasound knives are held in the right hand. Figure 1C, D). Irrespective of the development of the oblique fissure in the upper left lung, the principle of Export priority technique also applies to the anatomy of other single blood vessels.

### Export priority surgical procedure

Upon entering the chest cavity, the initial procedure involves examining the left upper lung's lesion location, assessing for adhesions in the left chest cavity, evaluating interlobular fissure development, and determining the presence of pleural dissemination and pulmonary metastasis. Surgical interventions encompass left upper lobe resection coupled with systemic lymph node dissection.

1. The oblique fissure, well-developed, mandates a sequential approach beginning with the opening of the interlobular fissure for addressing the left upper pulmonary artery. The progression is from shallow to deep, specifically targeting (A^4^ + A^5^)—(A^1+2^c)—(A^1+2^a + b)—(A^3^)—(V^1+5^)—B (^1+5^), accompanied by systematic hilar and mediastinal lymph node dissection. Adhering to the Export priority principle, initial attention is directed toward (A^1+2^a + b) and (A^3^), ensuring complete exposure of the left upper pulmonary vein Export before disconnection. Figure 2A, B). In the presence of a left upper mediastinal tongue artery, initiating the procedure from (A^1+2^c) is advisable. Choosing linear cutting staplers demands a strategic approach, favoring those that allow angle adjustments. In cases of elevated vascular tension or difficulty advancing the cutting stapler, treatment options include silk thread ligation and arterial clipping. Subsequent to disconnecting all branches of the pulmonary artery in the left upper lobe, attention is turned to addressing the left upper lobe pulmonary vein and bronchus.

**Fig. 2:**
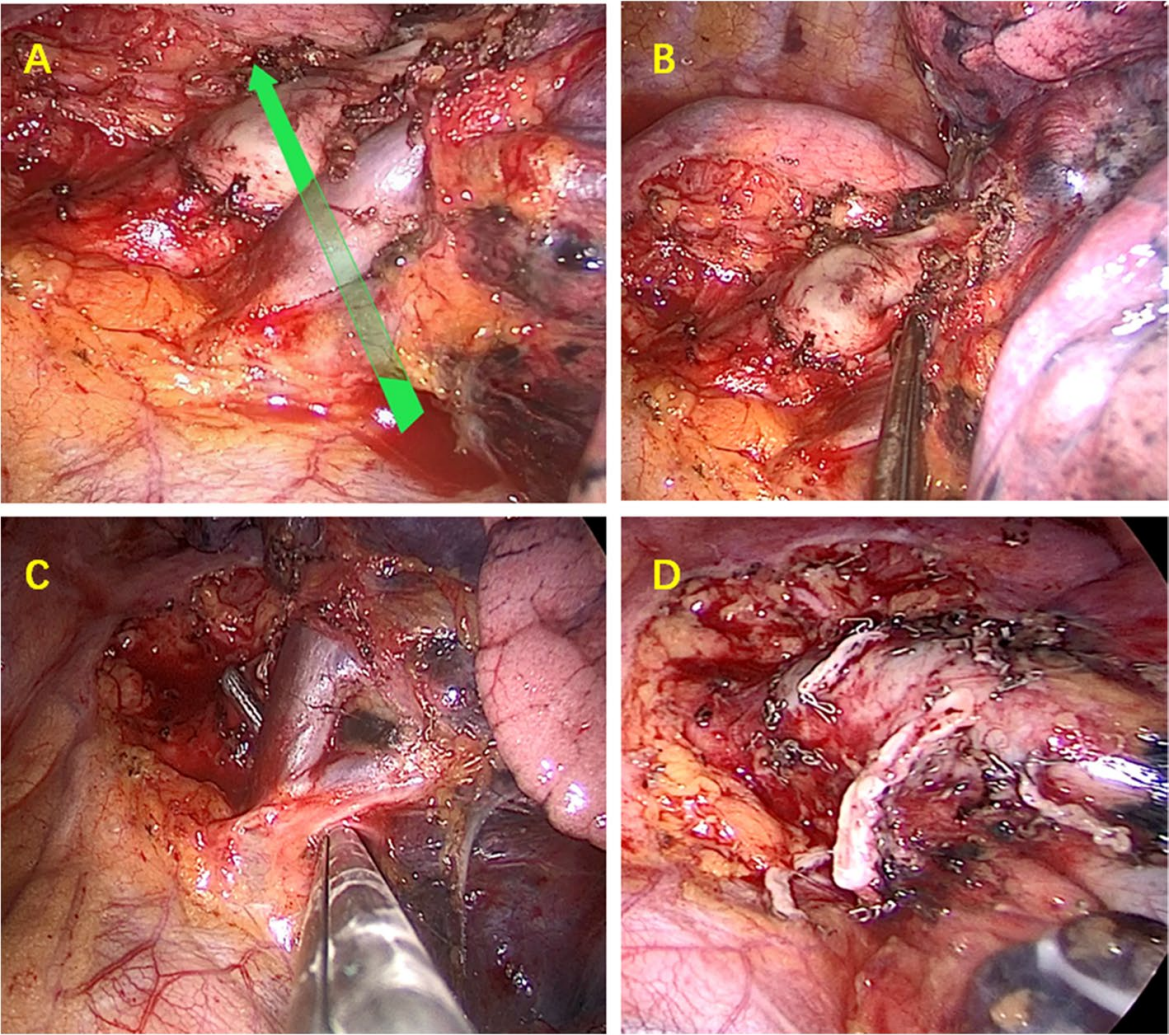
Application of export priority technology in Uni-portal thoracoscopic left upper lobectomy + lymph node dissection. **A** When dealing with veins first, arteries L(A^1+2^a + b) and LA^3^ block the posterior Export of the left upper pulmonary vein. Forcefully passing through the linear cutting occluder may damage the blood vessels and cause bleeding. **B** Fully free the left upper pulmonary artery and venous space, expose L(A^1+2^a + b) and LA^3^. **C** By utilizing export priority technology, LA^1+2^a + b and LA^3^ can be first incised to fully expose the left upper pulmonary vein Export. Cutting off the left upper pulmonary vein under Uni port conditions is easier and safer. **D** After the surgery, the condition of blood vessel and tracheal stumps
2. If the oblique fissure is poorly developed or undeveloped, it is essential to initially dissect away from the anterior mediastinal pleura to fully expose the left upper pulmonary artery trunk, (A^1+2^a + b), (A^3^), and the gap between the left upper pulmonary vein. Adhering to the principle of Export priority, prioritize handling L(A^1+2^a + b) and (LA^3^), open the Export, and subsequently disconnect the left upper pulmonary vein. The specific surgical sequence is as follows: L(A^1+2^a + b)—(LA^3^)—L(A^1+2^c)—L(A^4^ + A^5^)—L(V^1+5^)—L(B^1+5^) + systematic hilar and mediastinal lymph node dissection, Fig. 2C, D).

### Statistics

The statistical analysis data was processed using SPSS 26.0, and the quantitative data was represented by ($\overline{x}$ ± s). Comparison between two groups of data with normal distribution is conducted using t-test, while count data is conducted using t-test χ 2 Inspection. Inspection level a = 0.05.

## Results

Thirty-two patients who underwent Uni-portal thoracoscopy for radical surgery of left upper lung cancer were successfully treated, and all surgeries were performed following the principle of Export priority. Among them, there were 12 males and 20 females, with an average age of (52.53 ± 12.66) years. The total number of lymph node dissections was (13.96 ± 2.69) pieces, and the surgical time was (98.93 ± 14.98) minutes. The postoperative chest catheterization time averaged (3.1 ± 1.84) days, while the overall length of hospital stay was (5.62 ± 1.81) days. The average surgical bleeding volume was (79.53 ± 37.96) ml. The Day 1, chest tube drainage was (101.87 ± 49.46) ml. The postoperative pathological staging was predominantly distributed from stage I to stage IIIA, with 18 cases in stage I, 11 cases in stage II, and 3 cases in stage III. One patient developed chylothorax after surgery, and successful recovery was achieved through a fat-free diet, nutritional support, and one week of fluid replacement before discharge. There were no deaths during or within one month after surgery, and no transition to three-hole or traditional open chest surgery, Table 1.

## Discussion

UVATS left upper lobectomy is widely acknowledged as the most challenging procedure among all lobectomy surgeries, presenting the highest risk and likelihood of bleeding. This is primarily attributed to the intricate branching pattern of the left upper pulmonary artery, which is prone to variations, and the absence of a direct route for the left upper pulmonary vein and artery, both of which encircle the left upper pulmonary bronchus. Consequently, the complexity of Uni-portal thoracoscopic left upper lobectomy resection is significantly heightened. In the early stages, the absence of angled linear cutting staplers led many surgeons to believe that a Uni-portal approach was unsuitable for left upper lobectomy [5, 12]. Since Gonzalez Rivas first reported a case of UVATS left upper lobectomy in 2011 [13], Many thoracic surgeons have incorporated the Uni-portal approach into their surgical techniques [14, 15]. Over the past two decades, advancements in Uni-portal thoracoscopic technology, coupled with continuous innovation and instrument development, have contributed to the maturation of Uni-portal thoracoscopic surgery [16–18]. As a result, left upper lobectomy is no longer deemed a restricted procedure for Uni-portal applications [19–21]. In our study, we introduced a novel concept known as the "principle of Export priority." This principle emphasizes the strategic sequencing of procedures, particularly prioritizing the dissection of A^3^ and A^1+2^a + b before addressing the left upper pulmonary vein. This intentional sequencing, aimed at minimizing Export obstruction, streamlines the subsequent processing of the left upper pulmonary vein, effectively mitigating the risk of significant bleeding. After conducting propensity score matching for 544 cases of non-small cell lung cancer, Sun [22] concluded that Uni-portal UVATS and three-port VATS are equally safe and feasible. UVATS exhibited a median surgical time of 114 min. Studies by MU [23] revealed that the efficacy of UVATS lobectomy is comparable to that of three-port thoracoscopic lobectomy, with an average operating time of (138.83 ± 63.63) minutes. Wang et al. [24].'s research emphasized the influence of factors such as anesthesia, patient position, and instruments in UVATS left upper lung cancer radical surgery, reporting an average surgical time of (164.70 ± 12.50) minutes. Our study employs Export Priority technology to optimize vascular treatment, resulting in a significantly reduced surgical time of (98.93 ± 14.98) minutes, which is notably lower than reported in the literature [25]. HU's analysis [8] of 204 cases of UVATS revealed a median lymph node dissection of 12 (10,13) and a median postoperative hospitalization time of 5 (4,6) days, aligning closely with our research findings. Throughout the surgery, meticulous efforts were made to liberate both the anterior and posterior mediastinal pleura, ensuring clear exposure of the left upper pulmonary artery trunk. In instances of challenging left upper lobectomy or arterial bleeding, a proactive measure was taken by pre-blocking the left upper pulmonary artery trunk. Wang et al.'s research [26] indicates that a comprehensive dissection of the mediastinal pleura and the incorporation of the concept of broad exposure can enhance the efficiency of Uni-portal thoracoscopy lymph node dissection, providing better protection for crucial surrounding tissues and organs. This aligns with our perspective. When managing other pulmonary artery branches, we advocate for the applicability of the Export priority approach, especially within the limited field of view of a Uni-portal thoracoscope. Vascular inlet anatomy is more comprehensively visualized in our field of view, while Export anatomy often remains insufficiently exposed. This imbalance elevates the risk of unplanned bleeding during the passage of the cutting closure device. Ensuring parity between the visibility of the Export and entrance anatomy significantly enhances surgical safety. When investigating lymph node fusion in the chest cavity, it is essential to pre-block the pulmonary artery trunk while fully dissecting the pulmonary artery, both the inlet and outlet. This precautionary measure helps mitigate the risk of bleeding and ensures confidence for subsequent sleeve lung resection. A thorough incorporation of the Export priority concept holds the potential to diminish the risk of unplanned bleeding, reduce surgical duration, and equip surgeons to handle challenging procedures with greater composure.

Due to the improvement of modern living standards, most patients suffer from obesity [27, 28]. Excessive and thick subcutaneous adipose tissue accumulation on the surface of the ribs undoubtedly increases the difficulty for the surgeon to locate the ribs. We propose a new concept for precise rib positioning, which utilizes the principle of parallel correspondence between the lower corner of the shoulder armor and the 5th rib to quickly locate the rib gap. Accurate and perfect incision selection can effectively improve the comfort of surgeons and is also an important foundation for ensuring surgical safety. Triple port thoracoscopy often uses a 4th/7th/9th or 3th/7th/9th intercostal incision mode [22, 29], and the surgeon's right hand holding an electrocoagulation hook requires a longer path to dissect the blood vessels in the 9th intercostal space through the posterior axillary line, making the surgery itself difficult. The Uni-portal surgical approach may reduce the complexity and learning curve of left upper lobectomy, which requires further research to confirm, especially beneficial for right-handed dominant surgeons [30].

Our study introduces an innovative intercostal localization method designed specifically for patients facing challenges in localization due to obesity. Simultaneously, a retrospective analysis was conducted on data from 32 cases of Uni-portal thoracoscopic left upper lobectomy utilizing the export priority concept. The findings affirm the safety and reproducibility of our proposed Export priority concept. Nevertheless, our research does have limitations. Firstly, being a single-center study, it lacks the benefit of data feedback from multiple centers. Secondly, the study's sample size is relatively small, necessitating further research with an expanded sample size in the future.

In summary, Export priority technology has shortened surgical time, reduced unplanned bleeding, and may become an effective method for treating left upper lung lobectomy with UVATS.

## Data Availability

No datasets were generated or analysed during the current study.

## Abbreviations

UVATS: Uni-portal video-assisted thoracoscopic surgery
L(A^1+2^a + b): Left upper apical posterior segment artery (a + b) branch
LV^1^^−^^5^: Left upper pulmonary vein
LB^1^^−^^5^: Left upper pulmonary bronchus^1^^−^^5^
LA^3^: Left upper anterior pulmonary artery
BMI: Body Mass Index
COPD: Chronic obstructive pulmonary disease
FEV1: Forced expiratory volume in 1 s,
PO2: Partial pressure of oxygen
PET/CT: Positron emission tomography / Computed tomography
